# Adherence to option B+ PMTCT program and its predictors among HIV‐positive women in Ethiopia. A systematic review and meta‐analysis

**DOI:** 10.1002/hsr2.1404

**Published:** 2023-07-05

**Authors:** Habtamu Geremew, Demeke Geremew, Samuel Abdisa, Anteneh Mengist Dessie, Getachew Mullu Kassa, Nurilign Abebe Moges

**Affiliations:** ^1^ College of Health Sciences Oda Bultum University Chiro Ethiopia; ^2^ Department of Medical Laboratory Sciences, Immunology and Molecular Biology Unit, College of Medicine and Health Sciences Bahir Dar University Bahir Dar Ethiopia; ^3^ Department of Public Health, College of Health Sciences Debre Tabor University Debre Tabor Ethiopia; ^4^ Department of Public Health, College of Health Sciences Debre Markos University Debre Markos Ethiopia

**Keywords:** adherence, Ethiopia, HIV‐positive women, option B+

## Abstract

**Background:**

Previously, few studies investigated level of adherence to option B+ lifelong antiretroviral therapy (ART) in Ethiopia. However, their findings were inconsistent. Therefore, this review aimed to determine the pooled magnitude of adherence to option B+ lifelong ART and its predictors among human immune virus (HIV)‐positive women in Ethiopia.

**Methods:**

A comprehensive web‐based search was conducted using PubMed, Cochrane Library, Science Direct, Google scholar, and African Journals Online databases to retrieve relevant articles. STATA 14 statistical software was used to carry out the meta‐analysis. We used the random effects model to account for the large heterogeneity across included studies. Egger's regression test in conjunction with funnel plot and *I* ^2^ statistics were utilized to assess publication bias and heterogeneity among included studies respectively.

**Result:**

Twelve studies with a total of 2927 study participants were involved in this analysis. The pooled magnitude of adherence to option B+ lifelong ART was 80.72% (95% confidence interval [CI]: 77.05−84.39; *I* ^2^ = 85.4%). Disclosure of sero‐status (OR 2.58 [95% CI: 1.55−4.3]), receiving counseling (OR 4.93 [95% CI: 3.21−7.57]), attending primary school and above (OR 2.45 [95% CI: 1.31−4.57]), partner support (OR 2.24 [95% CI: 1.11, 4.52]), good knowledge about prevention of mother‐to‐child transmission (PMTCT) (OR 4.22 [95% CI: 2.02−8.84]), taking less time to reach health facility (OR 1.64 [95% CI: 1.13−2.4]), and good relation with care provider (OR 3.24 [95% CI: 1.96−5.34]) were positively associated with adherence. Whereas, fear of stigma and discrimination (OR 0.12 [95% CI: 0.06−0.22]) and advanced disease stage (OR 0.59 [95% CI: 0.37−0.92]) were negatively associated.

**Conclusion:**

The level of adherence to option B+ lifelong ART was suboptimal. Strengthened comprehensive counseling and client education on PMTCT, HIV status disclosure, and male partner involvement are important to eliminate mother to child transmission and control the pandemic.

## 1 BACKGROUND

Globally, there were around 4000 new human immune virus (HIV) infections per day in 2020; of which 60% were in Sub‐Saharan Africa and 10% were among children.^1^ More than 90% of all new pediatric HIV infections are due to mother‐to‐child transmission (MTCT).^2^ In Ethiopia, despite all efforts, the final vertical transmission rate including during breastfeeding remains high (15%).^1^

Option B+ prevention of MTCT (PMTCT) is among the four global strategies to eliminate MTCT of HIV.^3^ It underlines the universal provision of lifelong antiretroviral therapy (ART) for all HIV‐infected women irrespective of their world health organization (WHO) clinical stage or cluster of differentiation four (CD4) count.^4^ Yet, its effectiveness is highly determined by women's adherence to the ART regimen.^3^ Adherence to ART is crucial for improving and maintaining the health of the mother and her offspring.^3^, ^5^

Suboptimal adherence to ART is a recognized threat to the successful implementation of lifelong ART programs all over the world.^6^, ^7^ Poor adherence is more critical in resource‐constrained settings including Sub‐Saharan Africa.^8^, ^9^ A recent nationwide analysis in Burkina Faso found that 14% of pregnant and breastfeeding women were nonadherent to ART.^10^ Moreover, a meta‐analysis of east African studies also revealed that more than one‐fourth of women were nonadherent to option B+ PMTCT.^11^ In Ethiopia, the rate of poor adherence to option B+ PMTCT ranges from 5% to 32.7%,^12^, ^13^, ^14^ nonetheless there is a paucity of conclusive evidence at the national level.

Poor ART adherence leads to a higher risk of MTCT, less effective viral suppression, increased maternal HIV/AIDS‐related morbidity and mortality, and enhances the development of drug resistance.^7^, ^15^, ^16^ Women's adherence to ART is predicted by different factors like educational status, disclosure of HIV status, presence of social and family support, experience of drug side effects, fear of stigma, level of partner involvement, counseling, and knowledge about PMTCT.^11^, ^13^, ^17^, ^18^, ^19^, ^20^, ^21^ However, the importance of these determinants varies between studies.

Dependable evidence on adherence to ART and its determinants is compulsory for evidence‐based health care in mitigating the impact of the pandemic. In Ethiopia, few studies reported the magnitude of adherence to option B+ PMTCT program. However, they present inconsistent findings and failed to summarize the national adherence level. Hence, this study was conducted to determine the magnitude of ART adherence and its predictors among HIV‐positive pregnant and lactating women on option B+ PMTCT program in Ethiopia.

## 2 METHODS AND MATERIALS

### 2.1 Reporting and study protocol registration

This systematic review was reported according to the Preferred Reporting Items for Systematic Reviews and Meta‐Analyses (PRISMA) statement.^22^ To avoid unnecessary duplication of efforts, our study was registered in the International Prospective Register of Systematic Reviews (PROSPERO) database with protocol number, CRD42022335749.

### 2.2 Search strategy and information sources

A comprehensive web‐based literature search was conducted using PubMed, Cochrane Library, Science Direct, Google scholar, and African Journals Online databases to retrieve relevant articles. Besides, gray literature like Google and online University repositories (Addis Ababa University, Jimma University, Haramaya University, and University of Gondar) were scrutinized. Reference list of pertinent articles were also audited to identify additional studies. The search was conducted from June 1 to June 26, 2022. We utilized the following search string for searching in PubMed database; ((((((adherence[tw] OR compliance[tw])) OR (“Treatment Adherence and Compliance”[Mesh] OR “Medication Adherence”[Mesh] OR “Patient Compliance”[Mesh]))) AND (((Option B plus[tw]) OR (PMTCT[tw] OR “Infectious Disease Transmission, Vertical/prevention and control”[Mesh] OR prevention of mother to child transmission[tw]))) AND ((women) OR (“Women”[Mesh] OR “Pregnant Women”[Mesh] OR “Postpartum Period”[Mesh]))) AND Ethiopia.

### 2.3 Inclusion and exclusion criteria

This review included all primary studies conducted in Ethiopia and fulfills the following criteria.

Study area: studies conducted only in Ethiopia.

Study design: all observational studies (cross‐sectional, case controls, or cohort) that reported the magnitude and/or predictors of adherence to option B+ PMTCT during pregnancy, lactation, or both.

Language: articles that were published in the English language between June 2013 and June 26, 2022, were considered.

Population: HIV‐positive pregnant and/or lactating women who are on option B+ PMTCT.

Exposure: predictors of adherence to option B+ PMTCT. These are characteristics that may increase or decrease women's adherence to option B+ PMTCT, such as age, residence, educational status, disclosure of HIV status, drug side effect, and so forth.

Outcome: adherence to option B+ PMTCT.

However, editorials, duplicate studies, abstracts without full text, studies that were conducted before the option B+ era, and studies that reported adherence on nonpregnant and/or nonlactating women were excluded from this study. Besides, qualitative studies without the outcome of interest were also excluded from this analysis.

### 2.4 Outcome of interest

The magnitude of adherence to option B+ PMTCT among HIV‐positive pregnant and lactating women was our primary outcome of interest. There are several methods to measure adherence; commonly it can be measured by the self‐report method, pill count method, or else a combination of these methods can also be employed.^23^, ^24^, ^25^ Secondly, factors associated with adherence to option B+ PMTCT were identified.

### 2.5 Study selection, quality assessment, and data extraction

After removing duplicates, studies were screened based on their title and abstracts. For articles found to be relevant by title and abstract, a full‐text review against the specified inclusion/exclusion criteria was conducted to identify potential articles to be included in this review. We utilized reference management software (Endnote version X7.2); to combine database search results, remove duplicate entries and manage the citation process.

The Joanna Brigg's Institute (JBI) quality assessment checklist for prevalence studies was used to assess the quality of included studies.^26^ Two independent reviewers (H. G. and D. G.) assessed the quality of each study and inconsistency was resolved by involving a third reviewer (S. A.).

We used a standardized data‐extraction form which was developed considering the JBI guide for data extraction and synthesis.^27^ Records of primary studies were extracted by two independent authors. The abstracted data include primary author name and year of publication, region, study participants (pregnant or lactating women, or both), study design, sample size, number/prevalence of adherent women, and method used to measure adherence. Besides, data about all variables which were found to be significant predictors in one of the included studies and reported in at least one other study were extracted in separate excel spreadsheets.

### 2.6 Statistical analysis

Microsoft Excel spreadsheet was used to extract data from primary studies. Then, extracted data were exported to STATA version 14 for further statistical analysis. The *I* ^2^ statistics in conjunction with *p* values were used to assess heterogeneity between studies, and it was considered as low, moderate, or high when *I* ^2^ test statistics results were 25%, 50%, and 75% respectively.^28^ The pooled effect size was estimated using the random effects model (DerSimonian‐Laird method) to account for the large heterogeneity across included studies.^29^ The presence of publication bias was evaluated using funnel plot and Egger's regression test.^30^ A *p* value less than 0.05 was used to declare the presence of publication bias. Besides, sensitivity analysis was done to assess the influence of each study on the overall meta‐analysis estimate.

## 3 RESULTS

### 3.1 Identification and documentation of studies

**Figure 1:**
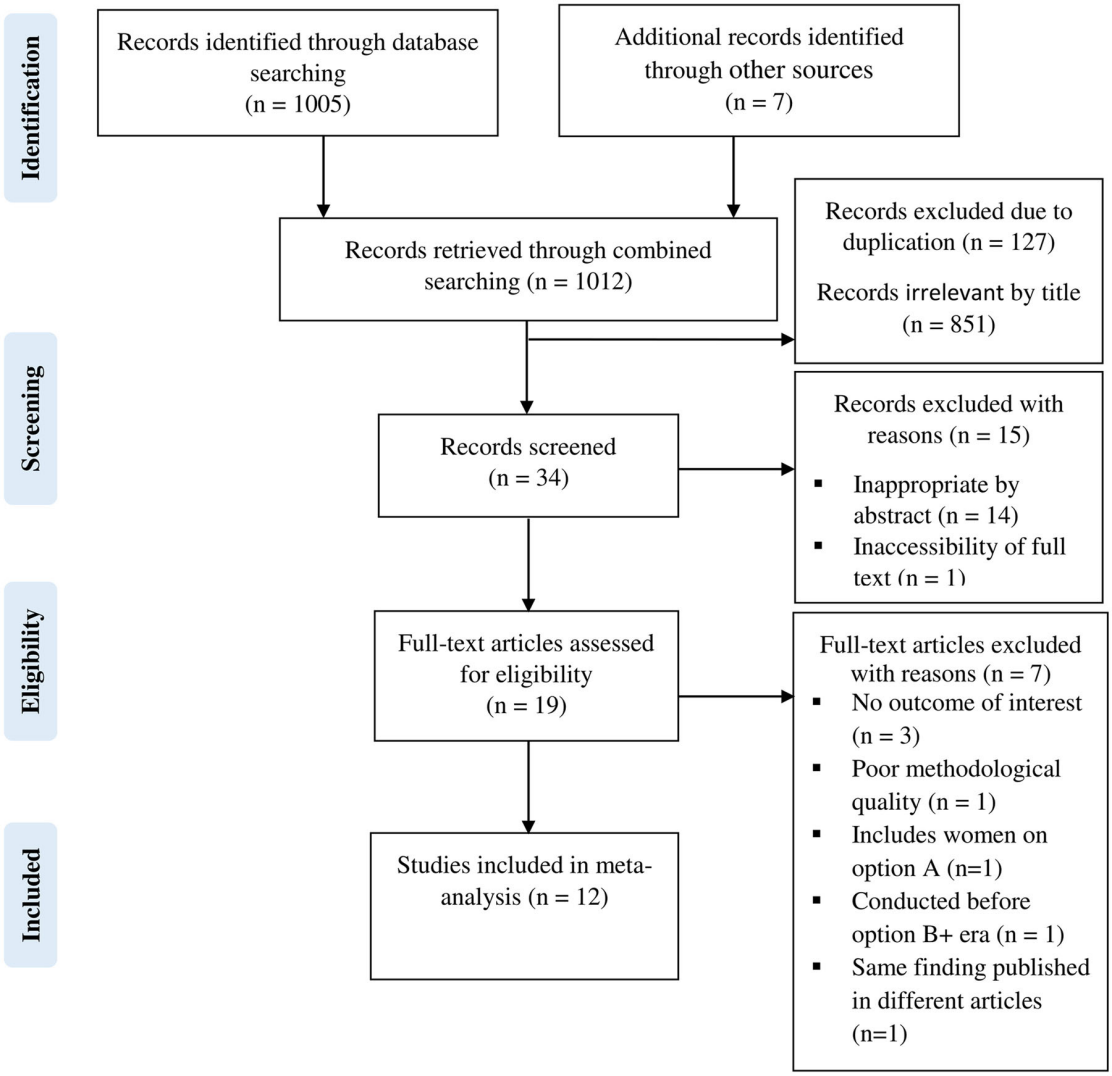
PRISMA flow diagram for the studies identified, screened, and included.

A total of 1012 studies were identified through the combined literature search. Of which, 127 duplicates were removed and 865 studies were excluded after screening by title and abstracts. One study was excluded due to the inaccessibility of the full text. Finally, the full texts of 19 studies were assessed for eligibility, and 12 studies were found to be appropriate for consideration in the quantitative meta‐analysis (Figure 1).

### 3.2 Characteristics of included studies

Twelve studies with a total of 2927 study participants were involved in this analysis. All included studies were cross‐sectional and conducted between 2014 and 2019. The sample size of included studies ranged from 103^31^ to 350.^14^ Six of the studies,^20^, ^31^, ^32^, ^33^, ^34^, ^35^ involved only HIV‐positive pregnant women whereas the remaining six studies,^13^, ^14^, ^19^, ^25^, ^36^, ^37^ included both pregnant and lactating mothers living with HIV. Adherence was measured by the self‐report method in eight studies.^13^, ^14^, ^19^, ^20^, ^32^, ^33^, ^34^, ^35^ Whereas the remaining four studies employed both self‐report and pill count methods to measure adherence.^25^, ^31^, ^36^, ^37^ Primary studies reported that adherence to option B+ PMTCT ranges from 66.02%^31^ to 88.19%^13^ (Table 1).

**Table 1:** Characteristics of studies included in the meta‐analysis.

Study and Year	Region	Participants	Method used	Study design	Sample size	Magnitude of adherence
Abdisa et al.^13^	SNNP^a^	Pregnant and lactating women	Self‐report	Cross‐sectional	254	88.19%
Asefa and Dirirsa^19^	Oromia	Pregnant and lactating women	self‐report	Cross‐sectional	180	81.11%
Demelash et al.^25^	Amhara	Pregnant and lactating women	Pill count and Self‐report	Cross‐sectional	269	75.09%
Ebuy et al.^32^	Tigray	Pregnant women	self‐report	Cross‐sectional	263	87.07%
Fedlu et al.^36^	Harari	Pregnant and lactating women	Pill count and Self‐report	Cross‐sectional	190	83.16%
Gebretsadik et al.^14^	Tigray	Pregnant and lactating women	self‐report	Cross‐sectional	350	67.43%
Suloro and Lodebo^35^	SNNP^a^	Pregnant women	self‐report	Cross‐sectional	202	83.66%
Aferu et al.^31^	SNNP^a^	Pregnant women	Pill count and Self‐report	Cross‐sectional	103	66.02%
Tarekegn et al.^33^	Oromia	Pregnant women	self‐report	Cross‐sectional	293	82.59%
Tesfaye et al.^34^	SNNP^a^	Pregnant women	self‐report	Cross‐sectional	290	81.38%
Tsegaye et al.^37^	Amhara	Pregnant and lactating women	Pill count and Self‐report	Cross‐sectional	190	87.89%
Wondimu et al.^20^	Oromia	Pregnant women	self‐report	Cross‐sectional	343	80.17%

*Note*: Keys: SNNPR.

^a^ Southern Nations, Nationalities and Peoples of Ethiopia.

### 3.3 Magnitude of adherence to option B+ PMTCT

**Figure 2:**
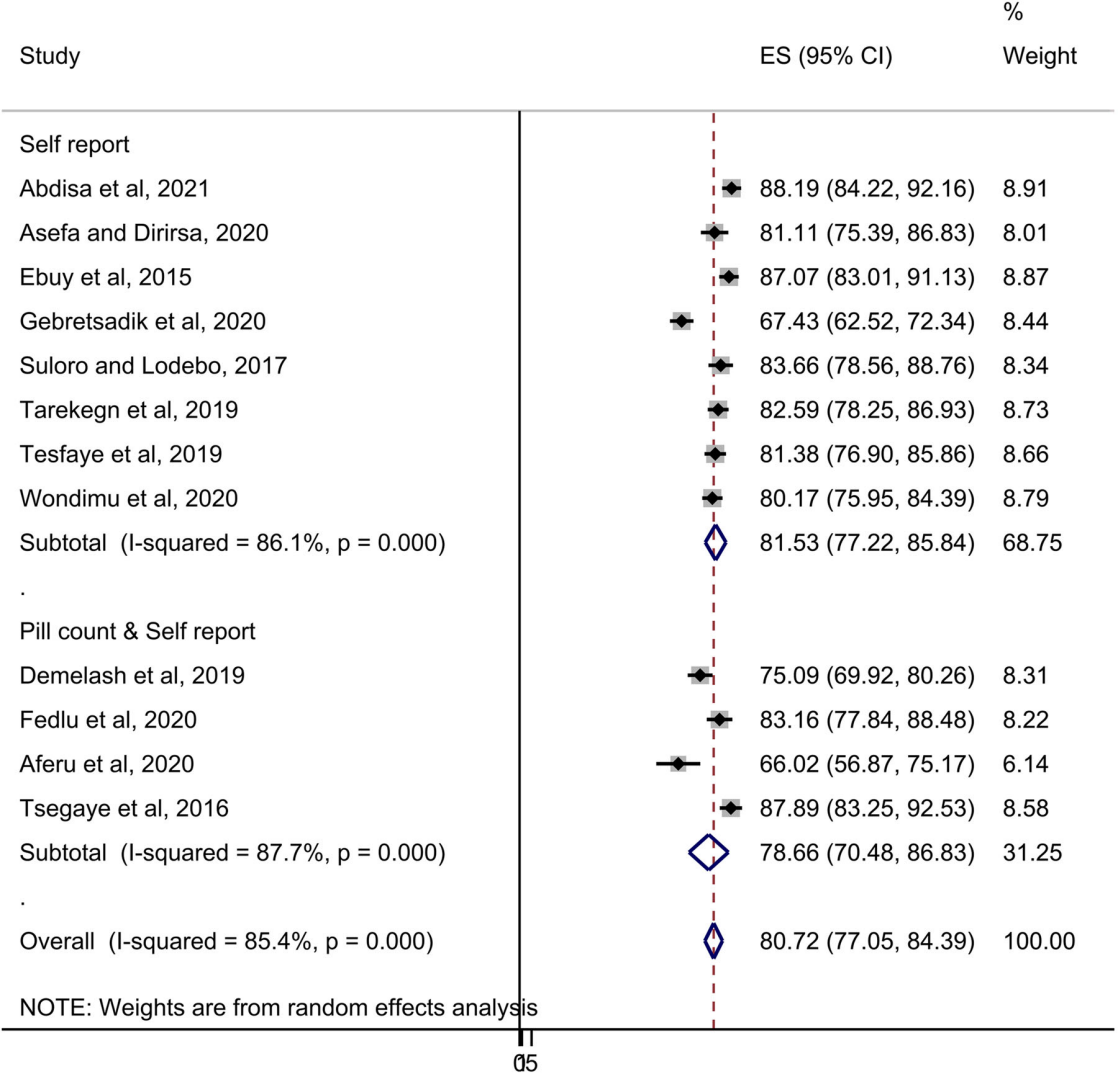
Pooled prevalence estimate of adherence to option B+ PMTCT in Ethiopia.

**Figure 3:**
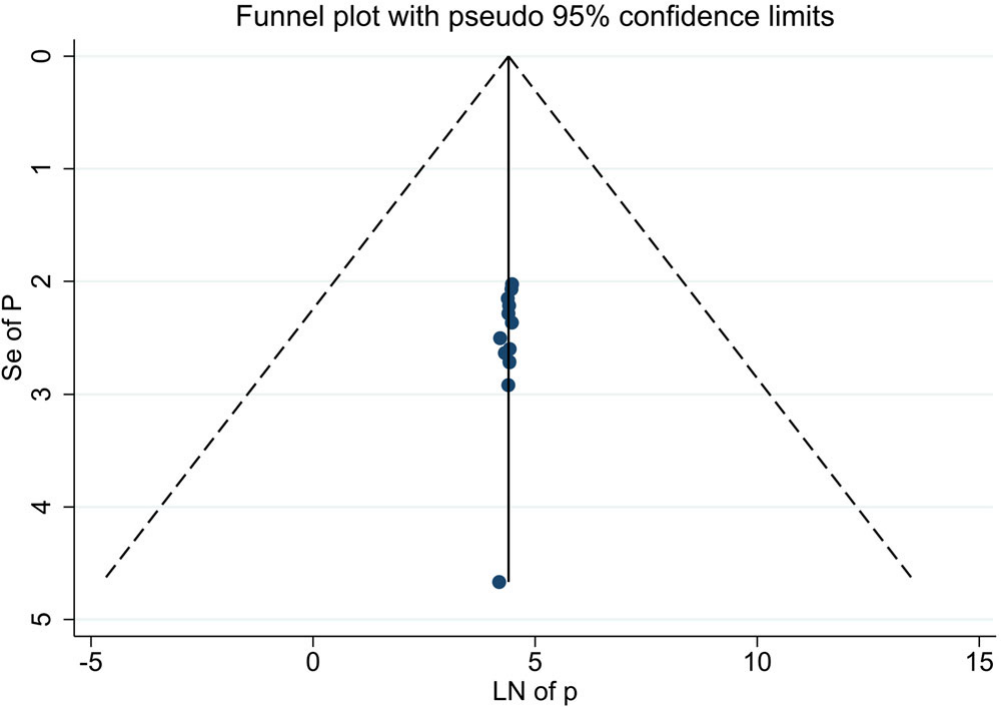
Funnel plot, evaluating the existence of publication bias for the pooled magnitude of adherence to option B+ PMTCT in Ethiopia.

The pooled magnitude of adherence to option B+ PMTCT in Ethiopia was 80.72% (95% CI: 77.05−84.39; *I* ^2^ = 85.4%). According to the sub‐group analysis by methods used to measure adherence, the magnitude of adherence among studies that used the self‐report method was 81.53% (95% CI: 77.22−85.84). Whereas that of studies that employed both self‐report and pill count methods was 78.66% (95% CI: 70.48, 86.83) (Figure 2). Egger's regression test indicated that there was no evidence of publication bias (*p* = 0.065). This is further corroborated by the symmetrical funnel plot (Figure 3). Moreover, sensitivity analysis showed that the effect of individual studies on the meta‐analysis estimate was not significant (Table 2).

**Table 2:** Sensitivity analysis, for the effect of each study on the summary estimate.

Study excluded	Estimate (95% CI)
Abdisa et al.^13^	80 (76.22−83.78)
Asefa and Dirirsa^19^	80.66 (76.70−84.62)
Demelash et al.^25^	81.23 (77.43−85.04)
Ebuy et al.^32^	80.10 (76.21−83.98)
Fedlu et al.^36^	80.47 (76.50−84.45)
Gebretsadik et al.^14^	82.17 (79.25−85.09)
Suloro and Lodebo^35^	80.43 (76.44−84.41)
Aferu et al.^31^	81.69 (78.15−85.24)
Tarekegn et al.^33^	80.50 (76.45−84.56)
Tesfaye et al.^34^	80.62 (76.58−84.67)
Tsegaye et al.^37^	80.05 (76.21−83.88)
Wondimu et al.^20^	80.74 (76.68−84.79)
Combined	80.72 (77.05−84.39)

### 3.4 Predictors of adherence to option B+ PMTCT

Data about 15 different variables were extracted in a two‐by‐two table and analyzed separately. Consequently, 10 variables were found to be significant predictors of adherence to option B+ PMTCT. Accordingly, women who disclosed their HIV sero‐status (OR 2.58 [95% CI: 1.55−4.3]), women who received counseling (OR 4.93 [95% CI: 3.21−7.57]), women who attended primary school and above (OR 2.45 [95% CI: 1.31−4.57]), women who reside in urban areas (OR 2.28 [95% CI: 1.51−3.43]), women who receive support from their partner (OR 2.24 [95% CI: 1.11−4.52]), women who had good knowledge about PMTCT (OR 4.22 [95% CI: 2.02−8.84]), women who need less than 1 h to reach the health facility (OR 1.64 [95% CI: 1.13−2.4]) and women who had good relation with care provider (OR 3.24 [95% CI: 1.96, 5.34]) were more likely to adhere to option B+ PMTCT. On the other hand, women who had fear of stigma and discrimination (OR 0.12 [95% CI: 0.06−0.22]) and women with advanced disease stage (OR 0.59 [95% CI: 0.37−0.92]) were having higher risk of nonadherence to the regimen (Table 3).

**Table 3:** Summary estimate of OR for factors associated with adherence to option B+ PMTCT in Ethiopia.

Variables	Intervention	Comparator	Included studies	OR (95% CI)	*I* ^2^
Disclosure of HIV status	Disclosed	Not disclosed	8	2.58 (1.55−4.3)	73.5%
Counseling	Counseled	Not counseled	8	4.93 (3.21−7.57)	52.1%
Educational status	Primary and above	No formal education	7	2.45 (1.31−4.57)	79.3%
Residence	Urban	Rural	8	2.28 (1.51−3.43)	63.2%
Partner support	Yes	No	9	2.24 (1.11−4.52)	87%
Knowledge of PMTCT	Good	Poor	6	4.22 (2.02−8.84)	80.1%
Fear of stigma and discrimination	Yes	No	2	0.12 (0.06−0.22)	0%
Time to reach health facility	<1 h	≥1 h	6	1.64 (1.13−2.4)	47.4%
WHO clinical stage at initiation	Advanced	Not advanced	3	0.59 (0.37−0.92)	0%
Relation with care provider	Good	Poor	3	3.24 (1.96−5.34)	0%

## 4 DISCUSSION

The period of pregnancy and lactation is marked by reduced adherence to lifelong ART which results in increased health risks to the mother and her child.^38^ This scientific inquiry was conducted to estimate the magnitude of adherence to option B+ PMTCT and identify its determinants among HIV‐positive women in Ethiopia.

As a result, the pooled magnitude of adherence to option B+ PMTCT in Ethiopia was 80.72% (95% CI: 77.05−84.39). This finding is lower than the WHOs' recommendation for optimal effectiveness of lifelong ART.^3^ This could be partly due to the impact of biological, social, and economic challenges associated with pregnancy and lactation.^39^ However, the estimate is higher than the finding of a previous meta‐analysis conducted in eastern Africa.^11^ The possible explanation for this variation could be the difference in number, setting, and design of included primary studies. Furthermore, despite the variation in the number of studies included, sup‐group analysis by methods employed indicated no significant difference in adherence level. This might suggest the effectiveness of all methods if used properly.^24^

Our meta‐analysis also identified determinants of adherence to option B+ PMTCT. Consequently, women who disclosed their sero‐status were 2.58 times more likely to adhere to the regimen as compared to women who did not disclose. This association is documented elsewhere,^11^, ^17^, ^40^ and could be because disclosing their sero‐status encourages women to ask for and receive support from their partners and/or families.^9^, ^40^ The odds of adherence were about five times higher among women who were counseled on the importance of PMTC and possible drug side effects than those women who were not. This finding was also revealed by previous reports.^11^, ^41^ The conceivable reason could be that counseling on the benefit of PMTCT and possible drug side effects increases clients' trust in health care providers and creates opportunities to clarify ambiguities.^42^

In line with previous studies, our analysis found that educational status was significantly associated with adherence to PMTCT.^9^, ^43^ Women who attended primary education and above were 2.45 times more likely to adhere to their lifelong ART regimen as compared to those women without formal education. This might be attributed to the reason that educated women had favorable attitude and perception towards PMTCT service.^44^ Similarly, women who reside in urban areas were 2.28 times more adhered than rural residents. This is consistent with the finding of previous studies.^45^ A likely explanation is that women from rural areas might have limited access to PMTCT services and information. Besides, poor educational status among rural residents might hinder their adherence to PMTCT.^46^

The odds of adherence were 2.24 times higher among women who received partner support as compared to those women who had not. This finding is congruent with findings of previous studies,^47^, ^48^ and could be attributed to improved psychological and economic accessibility by accompanying women to PMTCT clinics.^49^ Raised motivation and desire to have a healthy child, which is enhanced by partners' support could also be another reason. Likewise, women who had good knowledge of PMTCT were 4.22 times more likely to adhere to the regimen than their counterparts. This can be explained by the fact that good knowledge of PMTCT results in reduced misconception and a better understanding of the health benefit and effectiveness of PMTCT service.^50^ On the other hand, fear of stigma and discrimination reduces adherence by 88%. The possible explanation for this might be increased inconvenience due to the higher demand for confidentiality and the need not to be identified by others.^9^

This meta‐analysis also revealed that women who walked for less than 1 h to reach the health facility were 1.64 times more likely to adhere than women who walk for an hour or more. This could be attributed to the increased physical barrier and nontherapeutic cost associated with farther distances.^51^ Furthermore, adherence was 41% lower among women who had advanced disease (WHO clinical stage 3 and 4) compared to women with nonadvanced disease (WHO clinical stage 1 and 2). This might be explained by the reason that women with advanced disease might have additional drugs prescribed for coinfections, thus increasing the pill burden on them.^52^ Moreover, women who had good relationships with health care providers were 3.24 times more likely to adhere than their counterparts. This could be partly because good relationship fosters communication and facilitates individualized care.^53^

This study has certain limitations. Firstly, studies published only in the English language were considered. Some variables were not included because of variation in categorization across primary studies. Furthermore, all included studies were cross‐sectional therefore temporal associations cannot be established.

## 5 CONCLUSION

This meta‐analysis found that the level of adherence to option B+ PMTCT among HIV‐positive women in Ethiopia is suboptimal. Hence measures targeting the identified risk factors, like strengthened comprehensive counseling and client education about the purposes of PMTCT and possible drug side effects, HIV status disclosure, and male partner involvement are compulsory to eliminate MTCT and control the pandemic; with great emphasis on rural residents and poorly accessed women. Besides, universal primary education among women would have a substantial contribution to amplify the effectiveness of lifelong ART.

## AUTHOR CONTRIBUTIONS

**Habtamu Geremew**: Conceptualization; data curation; formal analysis; investigation; methodology; project administration; resources; software; validation; visualization; writing—original draft; writing—review and editing. **Demeke Geremew**: Data curation; formal analysis; investigation; methodology; project administration; resources; software; supervision; validation; visualization; writing—original draft; writing—review and editing. **Samuel Abdisa**: Data curation; investigation; methodology; project administration; resources; visualization; writing—review and editing. **Anteneh Mengist Dessie**: Data curation; investigation; methodology; project administration; resources; validation; writing—review and editing. **Getachew Mullu Kassa**: Investigation; methodology; project administration; supervision; validation; visualization; writing—review and editing. **Nurilign Abebe Moges**: Formal analysis; investigation; methodology; project administration; supervision; validation; visualization; writing—review and editing.

## CONFLICT OF INTEREST STATEMENT

The authors declare no conflict of interest.

## TRANSPARENCY STATEMENT

The lead author Habtamu Geremew affirms that this manuscript is an honest, accurate, and transparent account of the study being reported; that no important aspects of the study have been omitted; and that any discrepancies from the study as planned (and, if relevant, registered) have been explained.

## Data Availability

All data relevant to the study are included in the article.
